# Spatialization and Prediction of Seasonal NO_2_ Pollution Due to Climate Change in the Korean Capital Area through Land Use Regression Modeling

**DOI:** 10.3390/ijerph19095111

**Published:** 2022-04-22

**Authors:** No Ol Lim, Jinhoo Hwang, Sung-Joo Lee, Youngjae Yoo, Yuyoung Choi, Seongwoo Jeon

**Affiliations:** 1 Division of Environmental Science and Ecological Engineering, Korea University, Seoul 02841, Korea; limnori96@gmail.com (N.O.L.); i0255278@korea.ac.kr (J.H.); sungjoolee2@gmail.com (S.-J.L.); yeongjae7@gmail.com (Y.Y.); 2 Environmental Assessment Group, Korea Environment Institute, Sejong 30147, Korea; 3 Ojeong Resilience Institute, Korea University, Seoul 02841, Korea; cuteyu0@korea.ac.kr

**Keywords:** air pollution, land usage, spatial prediction, future scenarios, pollution management

## Abstract

Urbanization is causing an increase in air pollution leading to serious health issues. However, even though the necessity of its regulation is acknowledged, there are relatively few monitoring sites in the capital metropolitan city of the Republic of Korea. Furthermore, a significant relationship between air pollution and climate variables is expected, thus the prediction of air pollution under climate change should be carefully attended. This study aims to predict and spatialize present and future NO_2_ distribution by using existing monitoring sites to overcome deficiency in monitoring. Prediction was conducted through seasonal Land use regression modeling using variables correlated with NO_2_ concentration. Variables were selected through two correlation analyses and future pollution was predicted under HadGEM-AO RCP scenarios 4.5 and 8.5. Our results showed a relatively high NO_2_ concentration in winter in both present and future predictions, resulting from elevated use of fossil fuels in boilers, and also showed increments of NO_2_ pollution due to climate change. The results of this study could strengthen existing air pollution management strategies and mitigation measures for planning concerning future climate change, supporting proper management and control of air pollution.

## 1. Introduction

Today, air pollution, due to continuous urbanization, has become a global issue in both social and environmental spheres, especially since it is closely related to quality of life. Many studies have reported a relationship between the increasing rate of air pollution and the outbreak of air pollution-related diseases [1,2,3,4,5,6,7]. Additionally, the 2020 “annual update” report by the Air Quality Life Index (AQLI) shows that over the last two decades there has been little global reduction in air pollution, resulting in a two-year reduction in life expectancy per person globally [8].

According to the Organization for Economic Cooperation and Development’s (OECD’s) report on air pollution, the Republic of Korea is among the worst of the OECD member countries, suggesting that, by 2060, it will experience the highest air pollution increase among the surveyed OECD member countries. As evidence, of the total disease outbreaks reported on the annual report of health insurance in South Korea 19.5% belong to respiratory system diseases caused by air pollution [9]. Both the highest air pollution concentration and disease outbreaks were observed in densely populated and urbanized areas, such as metropolitan cities. The capital metropolitan area of South Korea (which includes Seoul, Incheon, and Gyeonggi provinces), is the world’s fifth largest metropolitan area, is the main metropolitan area of South Korea, and is home to approximately 50% of the national population. The air pollution concentration in this area is intensifying, resulting from increased emission rates of pollutants, such as nitrogen dioxide (NO_2_), ozone (O_3_), particulate matter, etc. Therefore, the management and reduction of air pollution is important, considering the risks it poses to human health, especially respiratory health.

Unlike ozone or particle pollution, people near major roads and urbanized areas are more susceptible to air pollutants such as NO_2_ [1,2,10,11,12], which are emitted from the ground-level burning of fossil fuels from vehicles, power plants, industrial sources, etc. NO_2_ converts into other substances, such as ozone, through chemical reactions when it disperses, thus its levels are appreciably higher in close proximity to pollution sources. It is, therefore, considered a good indicator for measuring direct pollution from traffic or neighboring industries [13,14]. Despite this fact, the number of monitoring sites is relatively limited in the context of the seriousness of the issue. Therefore, many studies worldwide, including those conducted in Europe, United States, and China [15,16,17,18,19,20], have developed various predictive models, such as kriging, dispersion models, and Land Use Regression (LUR) models, to overcome the quantitative deficiency in monitoring sites. The LUR model utilizes measured pollutant concentrations at monitoring sites as dependent variables and several geographic, climatic and topographic predictor data as independent variables to analyze their relationship through multiple linear regression [14,21], which is then used to predict the pollution concentration at unmonitored areas. The LUR model is able to capture small-scale spatial variations within air pollutants because it uses site-specific and neighbor data as input, which is important, since the suitability of the model depends more on the variability of the land characteristics captured by the sites than the number of monitoring sites itself [22]. Considering that approximately half of South Korea’s population lives in the capital metropolitan area, the relative number of monitoring sites for NO_2_ is too low to accurately represent the pollution rate [23]. Despite this limitation, there has been little application of LUR or other models in South Korea, where prediction of unmonitored areas is necessary to better understand and confront rising air pollution levels.

In our study, we focused on predicting the present NO_2_ concentration of the capital metropolitan city of the Republic of Korea based on seasonal LUR modeling, using existing monitoring sites, surrounding land usage data, and climate variables of the study area. We first used correlation analysis to determine the most suitable buffer size for each variable, and then selected the applicable variables for the regression model. The screening was divided into two steps: first, extracting variables that are highly correlated with NO_2_ concentration and second, eliminating variables highly correlated between the variables themselves. The extracted variables were used for stepwise regression and some variables were screened due to insignificant t statistics. The regression models for each season were conducted with the final variables for NO_2_ prediction. Additionally, we used climate change scenarios to predict future potential air pollution in the year 2070 using the developed models, in order to better understand future air pollution trends, due to climate change, and apply them to various mitigation plans and to decision making. The prediction of NO_2_ concentration at unmonitored sites through the LUR model can contribute to identifying and considering the main influencing factors in air pollution management and regulations.

## 2. Materials and Methods

### 2.1. Study Area and Monitoring Sites

**Figure 1:**
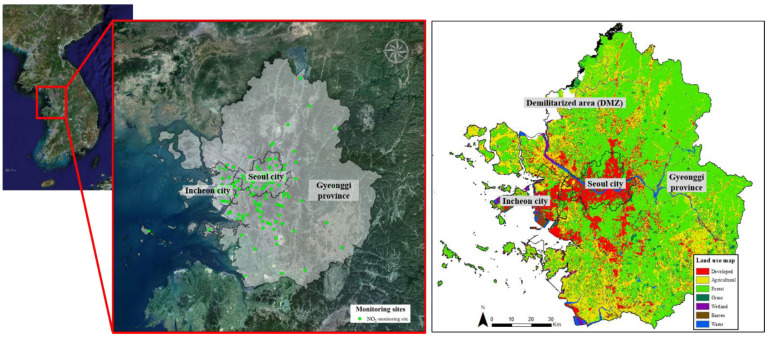
Study area. Location of the Capital metropolitan area (Seoul city, Incheon city, Gyeonggi province) with the 125 NO_2_ monitoring sites collected for 2018 (Airkorea), and Land use map for the study area.

The capital metropolitan area is the most populated and largest metropolitan area in the Republic of Korea. It includes the capital city, Seoul, as well as Incheon city, and the Gyeonggi province (Figure 1). It covers 11.8% (approximately 11,851 km^2^) of the entire national territory (100,410 km^2^), and accounts for more than 45% of the country’s total population, making it the fifth most largely populated metropolitan area in the world. Rapid urbanization and increasing overpopulation in the study area have drastically increased air pollution concentration into the 1990s, but according to the Air Pollution Management Department of the Ministry of Environment, the concentration of most pollutants has been decreasing since the 1990s, except for O_3_ and NO_2_, which have shown an increase and/or retention of previously measured values. Since the majority of NO_2_ emissions occur in highly urbanized and populated areas, we selected NO_2_ as the target pollutant of this study to clearly analyze the surrounding land usage impact and its relationship with the pollutant.

We collected real-time NO_2_ concentration data from a total of 125 NO_2_ monitoring sites (40, 30 and 55 sites for Seoul, Incheon, and Gyeonggi provinces respectively) across the entire study area from January to December of the year 2018, constructed by the Atmospheric Measurement Team of the Ministry of Environment (Airkorea). Monitoring sites should be settled at highly populated and developed areas for meaningful monitoring and management. The relatively few monitoring sites in Gyeonggi province is due to the large forest areas located all along the territory (Figure 1), making monitoring sites unnecessary, in contrast with Seoul capital or Incheon city where the majority of land consists of developed areas. Additionally, there are few or no monitoring sites in the northwest area, due to the presence of the ‘Demilitarized Zone’ (DMZ), which is the zone that demarcates North Korea from South Korea; an almost untouched and highly conserved area. Thus, the installation of monitoring sites is unnecessary and hazardous for resources within the zone.

Since seasonality intensely influences air pollutant emission and diffusion, in both anthropogenic and climatic aspects [24,25,26], we organized the collected concentration data into seasonal data by averaging the concentrations.

### 2.2. Predictor Variables

The variables used for the land use regression analysis were divided into five categories: traffic, land use, population, climate, and others. Each category was divided into several subcategories, as explained below (Table 1), and all variables were processed and rescaled to 30 m, using ArcMap 10.3.1. from Esri Inc. (Redlands, CA, USA).

#### 2.2.1. Traffic Variables

As vehicles are considered the primary source of NO_2_ emissions, traffic-related variables are important determinants of air pollution rate. Several studies have shown a high positive correlation between traffic-related variables and NO_2_ concentrations through land use regression modelling [13,14,27,28], and traffic variables were represented through diverse data, such as road length, traffic volume, etc. In our study, we used road length data collected from the Korea Transport Institute (KOTI, Sejong, Korea).

#### 2.2.2. Land Use and Population Variables

Data on land use variables, such as governmental, industrial, commercial, and residential areas, were collected from the national land cover map created by the Ministry of Environment (2018). To obtain the open space area as a variable in our study, we used the ‘urban planning facilities’ data from the Ministry of Land, Infrastructure, and Transport. These included national or county parks, public open facilities, and other areas considered in efficient land use management and planning.

The population density and housing density data were gathered from the national census of 2018 provided by the Korean Statistical Information Service, and were used to calculate the density of each buffer (refer to Section 2.3 for more information).

#### 2.2.3. Climatic Variables and Others

Monthly climatic data, including temperature, precipitation and wind speed, were collected from the Worldclim data website (Worldclim.org, accessed on 21 June 2020) and rescaled. The climatic data we used in this study were from 2018 and the future climatic data were from HadGEM2-AO under Representative concentration pathway (RCP) scenarios 4.5 and 8.5 for the year 2070. HadGEM2 family configurations of the Met Office Unified Model (MetUM) comprise configurations combining model components, which facilitate the representation of many different processes within the climate system. HadGEM2-AO is one of these combinations containing ocean and sea ice components, in addition to troposphere, land surface and hydrology and aerosols [29]. The National Institute of Meteorological Research under the Korea Meteorological Administration uses the HadGEM2-AO climate model when participating in the Coupled Model Intercomparison Project (CMIP5) long term experiments [30], thus we considered HadGEM2-AO model configuration appropriate for its application in South Korea as it is also widely used in other studies [30,31]. Additionally, RCP scenarios, reported on in the Fifth Assessment Report (AR5) by the Intergovernmental Panel on Climate Change (IPCC), show future greenhouse trends as a result of different regulation efforts, from high effort-low emission (4.5) to low effort-high emission (8.5). The collected climatic data were seasonally divided (Spring—March, April, May; Summer—June, July, August; Fall—September, October, November; Winter—December, January, February) and overlapped to calculate the seasonal climate value for both present and future data.

Altitude data was also collected from the Worldclim data website and distance to coast was calculated in meters measuring the distance between monitoring sites and coastline from the national land cover map created by the Ministry of Environment.

### 2.3. Processing and Screening of Variables

The Land Use Regression model is developed by first giving buffers to each monitoring site and extracting the surrounding land usage and climate data to verify their relationship with air pollution through linear regression modeling. Before proceeding to the regression modeling, we conducted a three-step analysis: one buffer size analysis and two correlation analyses.

First, we provided buffers with 100 m intervals, between 100 m to 1000 m, to every monitoring site to determine the most suitable buffer size, following previous studies [14,16,32], which applied several buffers of different sizes to detect the best fitting buffer size for each variable through correlation analysis between the buffers and the concentration at each site. This step is needed because the buffer size can influence each variable differently, depending on its characteristic or condition.

Once a suitable buffer for each variable was selected, we proceeded with the first correlation analysis. It was applied between the variables and monitored NO_2_ concentration to verify their relationship and delete variables showing low relationship with pollutant concentration. Finally, since variables with a high correlation between one another can influence the exactness or interpretability, we proceeded with a second correlation analysis among the variables selected from the first correlation analysis [14], and left the decisive variables for the regression analysis.

### 2.4. LUR Model Development

Once the decisive variables were obtained from the second correlation analysis, we performed regression modeling and analysis for each season to account for the influence of seasonality on air pollution. Stepwise linear regression is widely used for LUR modeling, as it automatically excludes variables with insignificant t statistics and develops adjusted regressions that can be readily applied to air pollution predictions [33,34,35]. Thus, we used the bidirectional stepwise linear regression method, which is a hybrid method of forward selection and backward elimination, using IBM SPSS Statistics 25 (Statistical Package of the Social Sciences) software. This stepwise regression method first incorporates strongly meaningful variables, based on the entry criterion of 95% confidence interval (*p* ≤ 0.05), and then excludes insignificant variables, based on the exclusion criterion of *p*-value > 0.1, repeating the steps simultaneously until no more significant incorporation or exclusion can be made.

After developing seasonal regression functions with the final variables, the accuracy of every function was verified through a k-folds cross validation (k = 10). K-fold validation divides the training set up into random subsamples, in our study 10 sets, from which one set is left out in turn as a validation set while the model calculates the regression on the remaining sets [36]. From the validation process we used the adjusted R^2^ and the Root Mean Square Error (RMSE) as the evaluating metrics for the model accuracy. Statistically, the model is considered accurate and adequate for application as the higher the R^2^ the lower the RMSE are. After verifying the validation of the regression, we applied and mapped the regression functions for a complete and elaborate NO_2_ concentration distribution, constructing a 30 × 30 m grid-map through ArcMap 10.3.1.

Additionally, we verified future NO_2_ concentration change due to climate change for both RCP 4.5 and RCP 8.5 scenarios. According to several studies and spatiotemporal land-use predictions [37], Korean land use and urbanization grew exponentially at the 1970s, but on entering the new millennium, population growth plateaued and may have begun to decrease, consequently resulting in limited or no land use change. Furthermore, the average annual percentage rate of urban change given by the UN World urbanization prospects for South Korea also showed the highest peak rate in the 1970s, which has decreased notably since the 2000s and maintained its rate with small fluctuations until the 2050s [38]. Therefore, we conservatively predicted future NO_2_ concentration across the study area considering only future climate change scenarios with the same variables used for the present regression analysis.

Standards for the national air quality of NO_2_, from the Korean Atmospheric Measurement Team and Korea Environment Corporation, suggest that the average yearly emission of NO_2_ should be less than, or around, 30 ppb in order not to impact on human health. Adopting this yearly 30 ppb standard as the threshold concentration, we divided the present and future seasonal and annual NO_2_ concentration distribution into low (lower than 30 ppb), medium (between 30 and 40 ppb) and high level (greater than 40 ppb) to verify the seriousness of seasonal and annual air pollution on human health.

## 3. Results

### 3.1. Processing and Screening of Variables

The optimum buffer sizes for each variable were obtained, as shown in Table 2, which were used for the first correlation analysis. The first correlation analysis was developed between the concentration from monitoring sites and every variable, to detect which variables are minimally related with concentration, so as to exclude them. As a result, housing density and distance to the coast showed low relationship with NO_2_ concentration and thus they were excluded. Thereafter, second correlation analysis proceeded between the variables left from the first correlation analysis, where population density showed high correlation with residential area, thus it was also eliminated and the remaining variables were used for stepwise regression. Once stepwise regression was developed, it automatically eliminated governmental and industrial areas, open space, wind speed and altitude as insignificant statistics, leaving road length, commercial and residential area, temperature and precipitation as the final variables. Hoek et al. (2008) reviewed land use regression studies worldwide and ordered the predictor variables collected from each study. Predictor variables used for NO_2_ prediction were those related with traffic (such as traffic intensity, road length, distance to road), population (such as population and household density, residential land cover), land use, physical geography and meteorology. The final variables of our study correctly reflect the relationship between NO_2_ pollution sources and its concentration, while also showing similar characteristics with the collected variables from other published studies mentioned before. Every correlation and regression analysis for climatic variables was developed seasonally, as mentioned before.

Additionally, among the final variables, the optimum buffer size for the traffic variable was relatively small (200 m) when compared to the residential area, which showed the largest optimum buffer size of 1000 m. Buffer sizes larger than 200 m for the traffic variable may incorporate sources too far away to be significant [39] and the small buffer size suggests that those living near roads are more vulnerable to pollutants than those living further away; meaning that near-road areas should be more sensibly managed when air pollution regulations are developed.

### 3.2. Seasonal Prediction and Spatialization of NO_2_ Concentration

The seasonal models had adjusted R^2^ values between 0.57 and 0.65, and RMSE values between 3.4 to 5.1 (Table 3), which were similar to those from previous studies [16,17,18,20,22,39,40]. Therefore, we considered the application of the models comprising the final variables and proceeded with stepwise linear regression analysis. As a result, road length showed the highest coefficient in every season during the whole year, explaining the high influence of traffic variables as the main pollutant source, highly correlated with NO_2_ concentration. Residential area also showed a high coefficient, coming after traffic variables in every season, according withprevious studies [15,41,42,43], where population related variables were selected as one of the significant predictors. This is because most of populated areas are densely urbanized, and, thusm are near pollution sources such as roads, and additionally, residential areas correspond to being one of the main sources of fossil fuel usage.

Temperature showed a similarly high coefficient as that of residential area in almost every season, especially in summer and winter, demonstrating parallel flow with previous studies [44,45,46], which showed a significant relationship between temperature and climate change with air pollution and the outcome of related diseases. However, regardless of the important interaction between climate and air pollution, only a few studies have considered climate variables as an important influencing factor when developing the LUR model. In further studies covering prediction of air pollution researchers should consider not only traffic and population variables but also climate-related variables for a more developed and effective prediction. Commercial area was not incorporated in the spring and summer final models due to the insignificant *p*-value (*p* > 0.05) obtained from the regression, as well as precipitation, which was excluded from summer and winter models.

#### 3.2.1. NO_2_ Concentration and Land Usage

**Figure 2:**
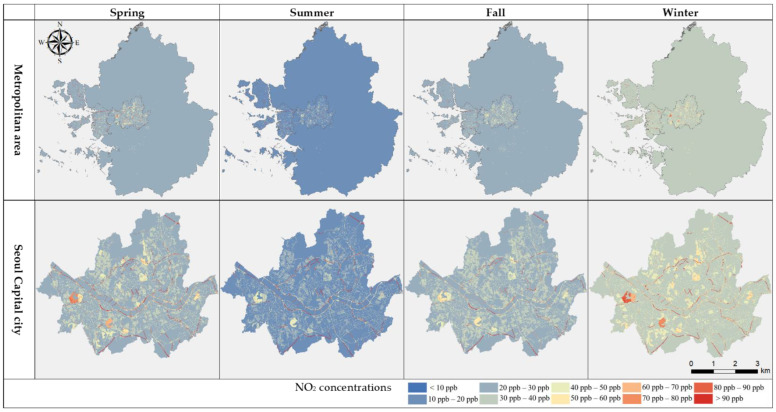
Present (2018) LUR seasonal models of the whole study area (Metropolitan area) and Seoul capital city.

The mapping results for present NO_2_ concentrations show a large concentration range over 50 ppb in Seoul capital city and minor parts of Incheon and Gyeonggi province (Figure 2). On the other hand, the majority of Gyeonggi province maintained a relatively low concentration range under 40 ppb during the whole year. The main difference between the capital city and other areas centers on different different land usage. The National Air Pollutant Emission Service reported that NO_2_ was the largest emitted air pollutant, with a total of 1,189,800 tons, and the main emission sources were traffic-related sources, followed by fossil fuel combustion (in both commercial and non-commercial areas, including energy industries), with 434,038 and 370,785 tons, respectively. The land use of Seoul capital city is mainly composed of residential and traffic areas (approximately 52.1%), which means that there is increase in combustion of fossil fuel for several human activities, such as the usage of boilers, transportation systems, commercial activities etc., and as the combustion rises so too do NO_2_ emissions. In contrast, Gyeonggi province, where there is a large percentage of mountainous terrain (approximately 54.4%), including the DMZ, intense urbanization is impeded, so there is only a small amount of the main pollutant sources. The importance of land usage and its impact on air pollution is very evident when comparing Seoul city and Gyeonggi province, making it clear that air pollution-related regulations should not only restrict pollutant emission sources like vehicles, but should also be concerned with sustainable land use management in preventative planning. The uniform and reasonable spatial distribution resulting from the LUR model developed in our study especially highlights the importance and applicability of the LUR modeling method in territories like Gyeonggi province, where the presence of monitoring sites is limited due to the terrain’s geographical characteristics.

**Figure 3:**
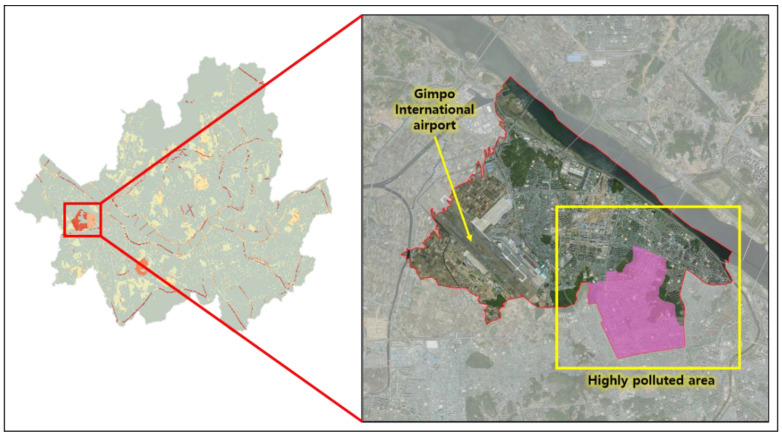
Satellite image of a highly polluted area (Gangseo-gu district).

Moreover, a particularly high NO_2_ concentration is visible on the western side of Seoul capital city (Figure 2), corresponding to Gangseo-gu district. Through satellite image analysis, the presence of Gimpo International airport near the highly concentrated residential area was observed, which occupies approximately 8.44 km^2^ of the total district area of 41.4 km^2^, rendering about 20% of the district’s territory inhabitable. According to the population statistics from the Big Data Department of Seoul Metropolitan Government, Gangseo-gu has become the second most populated district over recent years. Considering this fact, it can be implied that the density in residential areas in the district became relatively high (more than 15,000 per/km^2^) due to the restricted habitable space, causing over-population and thus over-usage of fuel combustion which leads to higher zonal NO_2_ concentration (Figure 3). This result shows the importance of understanding the relationship between different components related with air pollution in small-scale zones like districts and the necessity of verifying the main factor causing high pollution in addition to the pollutant itself, since zonal air pollution can be deeply affected not only by general factors, in this case traffic, but also by local characteristics like over-population and high density.

**Figure 4:**
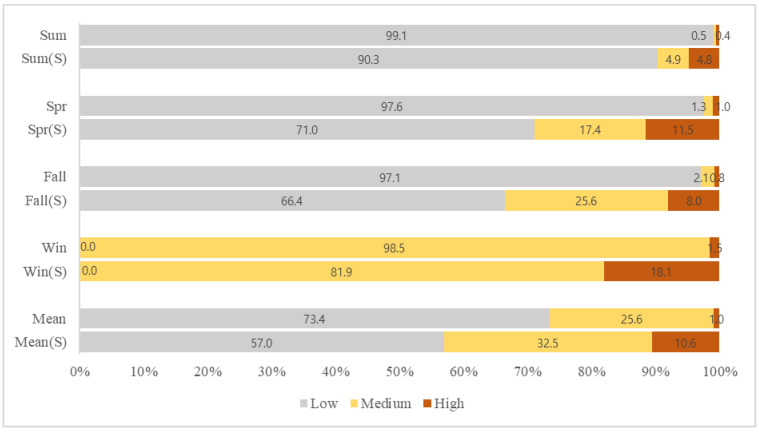
Mean and Seasonal present NO_2_ concentration level comparison at Metropolitan area and Seoul capital city (S = Seoul).

Standards for national air quality of NO_2_, from the Korean Atmospheric Measurement Team and Korea Environment Corporation, suggest that the average yearly emission of NO_2_ should be less than, or around, 30 ppb in order to not impact on human health. Adopting this yearly 30 ppb standard as the threshold concentration, we divided the seasonal and annual NO_2_ concentration distribution into low (lower than 30 ppb), medium (between 30 and 40 ppb) and high levels (greater than 40 ppb) to verify the seriousness of seasonal and annual air pollution on human health. Results showed that 25.6% of the overall study area has concentrations between 30 and 40 ppb (medium level), maintaining the annual national standard (Figure 4). In respect to seasonality, spring and fall models showed similar NO_2_ distribution, with approximately 97% of the concentrations being less than 30 ppb (low level) in most areas. Summer had the lowest annual concentration range, with approximately 99% of it being less than 20 ppb, in contrast with winter, where most concentrations were greater than 30 ppb.

Among the four seasons, summer showed the lowest emission range at 0.9%, followed by spring, fall and winter; the latter showing a high concentration range starting from 31 ppb. This result mirrored trends shown in previous studies [14,16,20,39], where high pollution levels were recorded in winter and explained by low precipitation levels and extensive use of heating. In effect, the concentrations in winter show parallel results with statistics of emission sources, as the usage of boilers increases in winter, thereby increasing the combustion of fossil fuels influencing NO_2_ emission concentrations. Wu et al. (2007) [47] analyzed seasonal emission characteristics from air polluting facilities, where the NO_2_ emissions from boilers during the winter were approximately 30 to 60 times greater than the emissions of the same pollutant in summer, emphasizing the higher levels of pollution in winter.

When only considering Seoul city, approximately 43% of the overall annual concentration appeared as either medium or high level, which is much higher than that of the whole study area. Additionally, even though only about 0.9% of the whole study area’s concentration was considered high level, Seoul city, in particular, showed an average of approximately 11% high level showing a relatively high concentration in every season, when compared with Incheon and Gyeonggi province; summer had the lowest range of 4.77% and winter the highest range of 18.06%, as seen in Figure 4. This trend shows the influence of greater use of fossil fuels due to different land usage and high population in the capital city, as mentioned before. According to the yearbook of regional energy statistics [48] the total energy consumption by sector in our study area totaled 57,898,000 tons. The ‘residential and commercial’ sector accounted for 35%, followed by the ‘transportation’ sector with 33%, and the ‘industry’ sector with 27%. In Seoul, the energy consumption of the ‘residential and commercial’ sector accounted for 55% of the total energy consumption, occupying more than half of total emissions, followed by ‘transportation’ and ‘industry’. The urban area percentage for the whole study area was about 10.5%, and that of Seoul city about 52.1%, and among these statistics 2.5% (total area) and 27.9% (Seoul city) of the territory is used for residential purpose. An important point is that the majority of NO_2_ emissions occur at residential areas which are affected by human activities determined by different climates, meaning that it can be controlled through some restrictions on human action. With increased awareness regarding issues caused by intense traffic and boiler usage in winter periods, several restrictions and mandates have been applied in metropolitan areas to reduce air pollution and overcome low air quality status. As an example, in 2020 the Korean government announced changes to the Special Act on Seoul Metropolitan Air Quality Improvement, mandating the installation of eco-friendly boilers with lower pollutant emissions. Simultaneously, in order to ameliorate fine dust emission, the fourth Special Committee on Fine Dust restricted the use of old diesel cars (identified as 5th class) and announced the ‘seasonal fine dust management implementation plan’. However, the application and execution of these new policies have been insufficient since they are broadly planned and are selectively focused on emerging particulate matter problems only. The seriousness of NO_2_ pollution is not yet adequately recognized. It also needs to be treated as an important and imminent health problem and should be the target of several seasonal management approaches, such as that for fine dust management.

#### 3.2.2. NO_2_ Concentration and Climate Change

**Figure 5:**
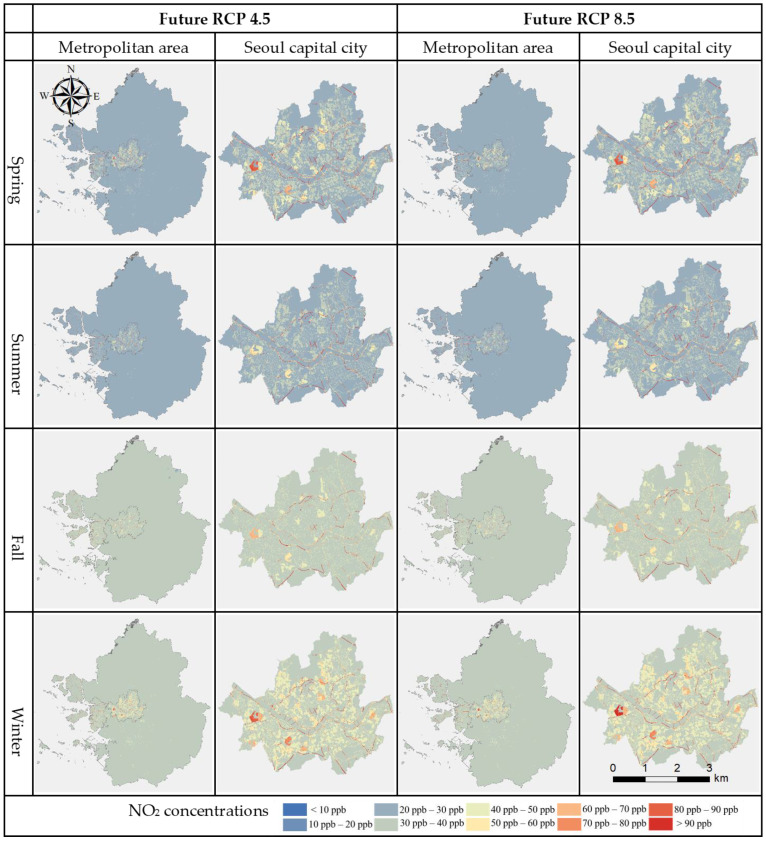
Future (2070) LUR seasonal models of the whole study area (Metropolitan area) and Seoul capital city under RCP scenarios 4.5 and 8.5.

Regarding the variables included in each seasonal model, climate variables and NO_2_ concentration rates demonstrated relatively high correlation, thus we carried out predictions of future air pollution due to climate change. Both RCP 4.5 and 8.5 scenarios showed a general increase in NO_2_ concentration in the entire study area for every season and especially in winter (Figure 5).

**Figure 6:**
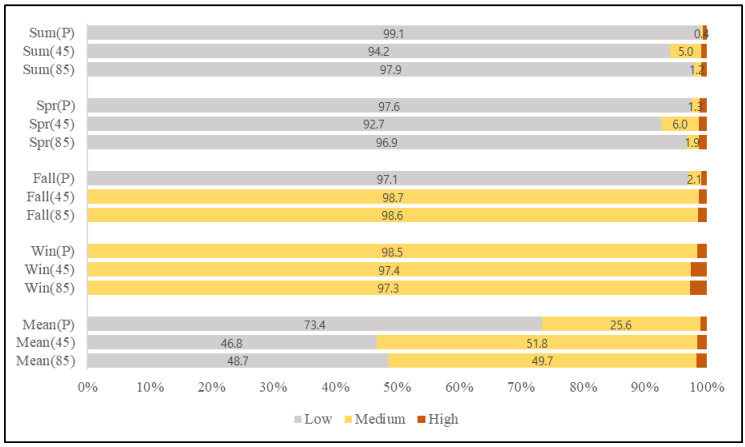
Mean and Seasonal NO_2_ concentration comparison for present and future scenarios at the whole study area (P = present; 45 = future scenario 4.5; 85 = future scenario 8.5).

For comparison with present concentrations, we divided the future prediction concentrations into the same levels as present results (low, medium, high). As a result (Figure 6), the low-level concentration for future prediction of RCP 4.5 and 8.5 scenarios consisted of 46.7% and 48.6% respectively, which indicate that in both scenarios the low-level areas decreased about 28% when compared with present low level concentration range occupancy of 73.4%. In contrast, medium level areas almost doubled from 25.6% in the present to 51.7% and 49.7% in RCP 4.5 and 8.5 scenarios, respectively. Finally, areas with high level concentrations increased from 0.9% in the present to 1.4% and 1.5% in RCP 4.5 and 8.5 scenarios, respectively (Figure 6).

These results show a general increase of NO_2_ concentration in the metropolitan area with future climate changes exposing higher health risks in the future. This incrementation could be related to the pollutant emission characteristics of fossil fuel usage in both residential and traffic arenas. First, increasing temperatures in summer and decreasing temperatures in winter influence human activities by increasing the usage of air conditioners or heaters in houses and cars, thereby affecting emissions of pollutants. Especially in the case of diesel cars, increased use of air conditioners affects exhaust gas recirculation (EGR), restraining its NO_2_ emission reduction function and resulting in a rapid increase of pollutant emissions [49]. This phenomenon will worsen when outside temperature, called the intake temperature, of vehicles increases with future climate change, especially in highly populated and developed cities like metropolitan areas. Here, the urban heat effect is also added to the higher atmospheric temperatures, resulting in greater pollutant emissions. Additionally, relatively high precipitation in the present summer period may also change due to climate change, resulting in minor ‘washing effect’ of air pollutants.

There were only minor changes in spring and summer concentrations between the present and future scenarios, with NO_2_ pollution remaining in the same range of around 30 ppb. Even though the concentrations in summer changed from low level to medium level of around 30 ppb, it still remained the season with the lowest pollution when compared to other seasons. Similarly, concentrations in winter also increased, but general pollution remained at a medium level. Fall showed the most significant difference, as it changed from medium to high level, exceeding the threshold concentration range that is considered highly risky according to country standards.

**Figure 7:**
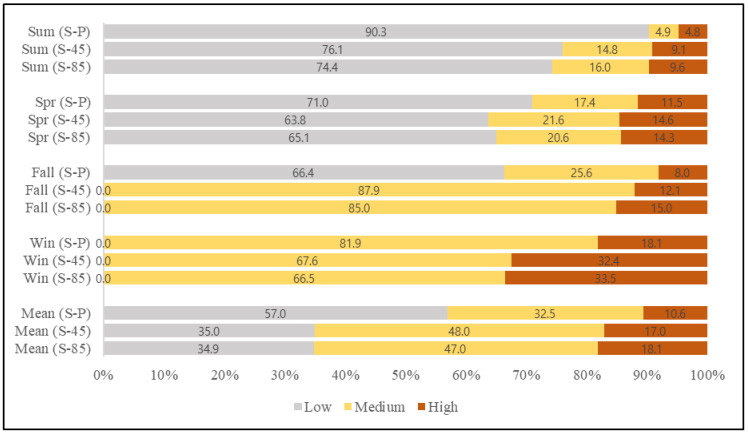
Mean and Seasonal NO_2_ concentration comparison for present and future scenarios at Seoul capital city (P = present; 45 = future scenario 4.5; 85 = future scenario 8.5).

In Seoul, the general changing trend from present to future was similar to that of the whole study area, but the changing scales were different (Figure 7). The annual average ratio for Seoul’s present NO_2_ concentration at low level was about 57%; summer showing 90.3% followed by spring and fall each showing 71.0% and 66.4%, respectively. This decreased to 35.0% and 34.9% for both RCP 4.5 and 8.5 scenarios, respectively. In the case of summer and spring, their ratios decreased to 76.1% and 63.8%, respectively, in contrast to those of fall and winter, which increased and exceeded the threshold of 30 ppb. Further, the areas pertaining to medium and high levels increased remarkably. The medium annual level increased from 32.5% to 48.0% and 47% for each scenario, and the high annual level increased from 10.6% to 17.0% and 18.1%, respectively. This increment was notable, especially in winter and fall, where the high-level areas of fall increased from 8.0% to 12.1% and 15.0% for RCP 4.5 and 8.5 scenarios, respectively. In winter the levels increased from 18.1% to 32.4% and 33.5% for each scenario (Figure 7).

Our study predicted the present NO_2_ concentration at unmonitored sites to provide a more accurate and developed model of pollution for more detailed decision-making. Over the course of the study, we also confirmed the relationship between climate change and NO_2_ pollutants through changes in human activities, due to climate change affecting pollutant emission source and land usage, which could threaten human health and lead to respiratory diseases, such as asthma, allergies, and acute pulmonary disease. To prevent the outbreak of such health problems, especially in the future when air pollution is expected to worsen, climate change prevention or mitigation measures should incorporate air pollution mitigation plans. Since the absolute prevention of air pollutant emissions is difficult, decision-makers should focus on prevention and mitigation measures. Once the importance and influence of land usage to air pollution-related diseases are recognized, it should be taken into account for elaborate land use planning that considers possible environmental and social damage. Additionally, there remains a need for mitigation policies protecting socially vulnerable groups exposed to air pollution and future climate change.

The limitations of this study mainly include the limitation of traffic data and the exclusion of future land use change predictions. First, the use of traffic density data is recommended, if available, but since specific traffic data was not given, we replaced it by road length, following similar previous studies.

Furthermore, as only low general land use changes were expected across the entire study area, according to several studies, including the UN prospects report, we excluded a land use change prediction for future air pollution predictions and focused on potential possible change due to climate change across the whole metropolitan area. However, when more detailed regional and site-specific predictions are needed, land use changes can affect future air pollution changes, even though those changes are small. Therefore, for a more accurate forecast of future air pollution in small scale studies, several scenarios, such as climate change, land use change, and socio-economic scenarios should be applied.

Finally, though we were able to show that NO_2_ emissions and climate change have a positive relationship in our study, the exact and specific cause of this relationship was not determined. Further studies should investigate NO_2_ and climate change interactions in greater detail, including chemical reactions and future land use trends to enable more specific decision making for the future.

## 4. Conclusions

In this study, we used NO_2_ monitoring sites to develop seasonal land use regression (LUR) models for air pollution predictions in the capital metropolitan area of South Korea, and also used future climate change scenarios to predict air pollution trends. Results showed that the present concentrations of seasonal NO_2_ pollution were highest in winter, followed by fall, spring, and summer. Statistical data and previous studies prove that fossil fuel combustion, due to considerable boiler usage increases during the winter, result in increased pollutant emissions. Seoul, in particular, showed the highest range of concentrations within the study area, as it is the most populated and urbanized area in the country. These results suggest the importance of nearby land use, and strengthen the necessity of land use and population dispersion management plans for air pollution sources.

The same seasonal trend was shown with future NO_2_ pollution predictions, but the gaps between seasonal ranges were narrowed down, indicating that, with increasing temperature and diversified precipitation, there were greater concentration changes and pollution rates in other seasons. This can be explained by changes in human activities when temperature increases, as increments in air conditioner usage in cars raise NO_2_ emissions and restrict the pollutant-treating function of diesel cars.

The specific present and future air pollution predictions in this study can support the strengthening of existing air pollution-related management, fossil fuel combustion regulations, and vehicle restrictions. The results also indicate the importance of land use regulation and seasonal management for mitigation of air pollution for future climate change planning.

## Figures and Tables

**Table 1:** Categories and Subcategories of Predictor variables collected. Every variable corresponds to the year 2018 and was rescaled into spatial resolution of 30 m.

Category	Subcategory	Measure Unit	Source
**Traffic** **variables**	Road length	Meters (m)	Korea Transport Institute (KOTI)
**Land use variables**	Governmental area	Square meters (m^2^)	Ministry of Environment (2018)
	Commercial area		
	Residential area		
	Industrial area		
	Open space		Ministry of Land, Infrastructure, and Transport
**Population variables**	Population density	Hab/area (m^2^)	Korean Statistical Information Service (2018)
	Housing density	Number of houses/area (m^2^)	
**Climate** **variables**	Temperature	Celsius degree (°C)	Worldclim (2018 and 2070)
	Precipitation	Millimeters (mm)	
	Wind speed	Meters/second (m/s)	
**Others**	Altitude	Meters (m)	Worldclim
	Distance to coast	Meters (m)	Ministry of Environment

**Table 2:** Screening process of final variables. The optimum buffer sizes for each variable (subcategory) were selected through buffer analysis. First correlation analysis (between NO_2_ concentration and the variables) and second correlation analysis (between the variables themselves) were conducted to screen the decisive variables. Final variables were selected through automatic exclusion and stepwise regression.

Category	Subcategory		Buffer Analysis	First and Second Correlation Analysis	Stepwise Regression—Automatic Exclusion
**Traffic variable**	Road length (road)		200 m	O	O
**Land use variables**	Governmental area (gov)		900 m	O	-
	Commercial area (com)		100 m	O	O
	Residential area (res)		1000 m	O	O
	Industrial area (ind)		100 m	O	-
	Open space (ops)		700 m	O	-
**Population variables**	Population density (pop)		1000 m	-	-
	Housing density		1000 m	-	-
**Climate variables**	Temperature	spring	1000 m	O	O
		summer	400 m		
		fall			
		winter			
	Precipitation	spring	100 m	O	O
		summer			
		fall			
		winter			
	Wind speed	spring	100 m	O	-
		summer	800 m		
		fall	100 m		
		winter	100 m		
**Others**	Altitude		500 m	O	-
	Distance to coast		-	-	-

**Table 3:** Seasonal LUR model results. Constant and coefficient of variables with significant *p*-value for the seasonal models and the model validation metrics (adjusted R^2^ and RMSE).

	Constant	Road	com	res	temp	prec	Adjusted R^2^	RMSE
**Spring**	0.023	5.9 × 10^−5^	0	3 × 10^−7^	0	1.8 × 10^−5^	0.582	5.1
**Summer**	0.015	5.5 × 10^−5^	0	1.8 × 10^−7^	3.3 × 10^−5^	0	0.648	3.4
**Fall**	0.025	3.7 × 10^−5^	1.4 × 10^−7^	1.7 × 10^−7^	2.6 × 10^−5^	2.8 × 10^−5^	0.569	5.1
**Winter**	0.031	4.3 × 10^−5^	1.6 × 10^−7^	3.1 × 10^−7^	3.3 × 10^−5^	0	0.611	4.3

Road: road length; com: commercial area; res: residential area; temp: mean temperature; prec: mean precipitation. Variables with *p*-value < 0.05 selected from the stepwise linear regression modelling.

## Data Availability

Not applicable.
